# Low prevalence of *LRAT* mutations in patients with Leber congenital amaurosis and autosomal recessive retinitis pigmentosa

**Published:** 2007-04-05

**Authors:** Meredith O. Sweeney, Terri L. McGee, Eliot L. Berson, Thaddeus P. Dryja

**Affiliations:** Ocular Molecular Genetics Institute and the Berman-Gund Laboratory for the Study of Retinal Degenerations, Harvard Medical School, Massachusetts Eye and Ear Infirmary, Boston, MA

## Abstract

**Purpose:**

To determine the the prevalence of pathogenic mutations in the gene encoding lecithin retinol acyltransferase (LRAT) in patients from North America with either Leber congenital amaurosis (LCA) or autosomal recessive retinitis pigmentosa (ARRP).

**Methods:**

Exon 1, exon 2, and the coding region of exon 3 of *LRAT* were PCR-amplified and directly sequenced from the leukocyte DNA of 82 unrelated patients with LCA and 190 unrelated patients with ARRP.

**Results:**

One isocoding change was found in this screen of *LRAT* (Glu114 GAG>GAA; c.342), and 5 other sequence changes were found in intronic or untranslated regions of the gene. None of these changes were predicted to affect the encoded protein and were therefore deemed non-pathogenic.

**Conclusions:**

*LRAT* mutations are likely a rare cause of LCA among patients from North America.

## Introduction

Lecithin retinol acyltransferase (LRAT), a 25.3 kDa protein found mainly in the retinal pigment epithelium and liver, catalyzes the formation of retinyl esters from all-trans retinol [1]. In the visual cycle, these retinyl esters are converted to 11-cis retinol by the isomerase activity of RPE65 [2]. The 11-cis retinol is subsequently converted to 11-cis retinaldehyde which is transferred to the photoreceptors where it serves as the chromophore in rod and cone opsins [3].

Two groups have reported a total of four patients with *LRAT* mutations. One found a missense mutation, Ser175Arg, in two unrelated homozygote patients with recessive early-onset severe retinal dystrophy [4]. Analysis of the corresponding mutant LRAT protein expressed in vitro showed that it had greatly reduced enzyme activity [4]. The same paper also described an obviously null frameshift mutation (396delAA) in a third patient who was a heterozygote with no mutation discovered in the other allele [4]. The second group found a patient with Leber congenital amaurosis (LCA) posessing a homozygous frameshift mutation (c.217_218delAT), which results in an early stop codon and likely would encode a nonfunctional protein [5]. Support for interpreting these null mutations as the causes of the patients' retinal degeneration comes from the analysis of transgenic mice lacking this gene; these mice have shortened photoreceptor outer segments and early loss of rod and cone photoreceptor function [6,7].

The term "early-onset severe retinal dystrophy" [4], can refer to either LCA or severe autosomal recessive retinitis pigmentosa (ARRP). Both LCA and ARRP are genetically heterogenous and many of the responsible genes remain unidentified. In order to better determine the proportion of patients with LCA or ARRP having mutations in *LRAT*, we performed a survey of patients with the clinical diagnosis of LCA and ARRP to find the prevalence of *LRAT* mutations associated with these two retinal diseases.

## Methods

This study conformed to the Declaration of Helsinki. We evaluated leukocyte DNA from 82 unrelated patients with LCA and 190 unrelated patients with ARRP. Our patients with LCA typically had nystagmus and visual acuities less than 20/200, while those with ARRP typically had no nystagmus and vision better than 20/200 at the initial visit. Many of these patients had been previously evaluated for mutations in the *PDE6A* [8], *PDE6B* [9], *CRX* [10], *RPE65* [11], *ELOVL4* [12], *CNCG1* [13], *USH2A* [14], *NRL* [15], *RPGRIP* [16], *USH3A* [17], *RHO* [18], *RDS* [19], *RDH5* [20], *CRALBP* [21], and *RGR* [22] genes; patients with definitely pathogenic mutations in one of those genes were excluded from this study. For each patient, the coding region of *LRAT* along with flanking intronic sequences, the entire 5' untranslated region (UTR), and 129 bp of the 3' UTR were PCR-amplified using 4 pairs of primers (Table 1). The amplicons were directly sequenced in one direction. Any sequence changes found were confirmed by sequencing in the opposite direction and were evaluated for their likelihood in altering splicing of the *LRAT* transcript using splice site prediction software found at the web site NNSPLICE 0.9 version [23].

**Table 1:** Primer sequences.

**Exon**	**Direction**	**Sequence (5'-3')**
1	Sense	CTCGACGGCCATAAAAAGTC
1	Antisense	AAAGACACCACCTCCAGCAT
2a	Sense	TACTTTGCGCCGTACCTCAC
2a	Antisense	GTAGGCGAAGTCCTCCACTG
2b	Sense	GAAGGTGGTCTCCAACAAGC
2b	Antisense	GGGGAAGAGAAAAGGTCAGG
3	Sense	CAGAAAATAGCTGGGAAAACTGA
3	Antisense	AAGCACTTTGCGTGATTCCT

The coding regions of lecithin retinol acyltransferase was polymerase chain reaction amplified from the leukocyte DNA of 82 unrelated patients with Leber congenital amaurosis and 190 unrelated patients with autosomal recessive retinitis pigmentosa using the 4 pairs of primers shown in this table.

## Results

We found six sequence variants in *LRAT* in eleven ARRP patients and two LCA patients (Table 2). Most of the changes were in introns or in untranslated regions of the exons. The only change in the coding region was an isocoding change in codon Glu114 (GAG>GAA; c.342) found in two LCA patients (all heterozygotes) and in five ARRP patients (4 heterozygotes and one homozygote), for a rare allele frequency overall of 1.5%. Splice site prediction software indicated that none of these changes were likely to alter RNA splicing (the splicing index was changed less than or equal to 0.01 compared to the normal sequence).

**Table 2:** Sequence changes found in *LRAT*.

**Exon**	**Sequence change**	**Number of heterozygotes (patient IDs)**	**Number of homozygotes**
1	5'UTR-15C>T	2 ARRP (003-147, 003-185)	0
1	IVS1+7A>C	2 ARRP (003-069, 003-161)	0
1	IVS1+13G>T	1 ARRP (003-178)	0
1	5'UTR-156C>G	1 ARRP (003-182)	0
2b	Glu114Glu c.342G>A	4 ARRP (003-089, 003-197, 003-285, 003-371)	1 ARRP (003-228)
		+ 2 LCA (048-020, 048-065)	
3	3'UTR+3T>G	1 ARRP (003-064)	0

Six sequence changes in *LRAT* were found among 11 autosomal recessive retinitis pigmentosa patients and 2 Leber congenital amaurosis patients.

## Discussion

Of the 6 sequence changes we found in *LRAT*, only one was found in the coding region, and that was the isocoding change in Glu114. Another group found this change in one ARRP patient, in one patient with macular degeneration, and in one patient with cone-rod dystrophy, all heterozygotes [24]. Furthermore, that same study found Glu114Glu in an unaffected homozygote [24]. Three previous studies by other groups also found the Glu114Glu change [4,5,25]. It was interpreted as a nonpathogenic polymorphism because it was found in 2.5% of alleles in retinal dystrophy patients, 2.7% of alleles in RP and flecked retinal dystrophy patients, and 3% of alleles in LCA patients analyzed; these allele frequencies are similar to the frequency of 1.5% found in our survey [4,5,25]. (Note: in the paper by Perrault et al. [25], this polymorphism was incorrectly labeled as Ser175Pro (unpublished communication from the authors)). The other changes we found are also unlikely to be pathogenic since they occur in introns away from consensus splice donor and acceptor sites or in the non-coding regions of exons; none is predicted to affect splicing.

**Figure 1:**
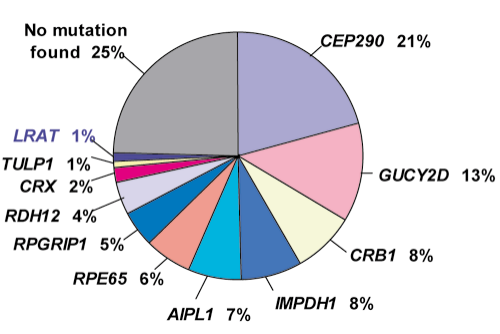
Estimated proportions of patients with Leber congenital amaurosis caused by the 11 identified Leber congenital amaurosis genes. This figure uses overall frequencies from Table 3. Eleven identified genes are responsible for approximately 75% of Leber congenital amaurosis cases; the gene(s) responsible for the remaining 25% of cases remain(s) unknown. CEP290 is responsible for the greatest number of cases (21%). The proportion of cases caused by *LCAT* mutations has been arbitrarily set at 1% (see Discussion).

Table 3 summarizes the results from 30 published studies (including this one) that describe searches among nine or more patients for mutations in eleven genes known to cause LCA (or early-onset retinal dystrophy which we have grouped together with LCA for this table). The two genes that account for the largest proportions of cases are *CEP290* (21% of cases overall) encoding a centrosomal protein and *GUCY2D* encoding guanylate cyclase (13% of cases). Figure 1 is a pie chart with the estimated proportions of LCA patients caused by each gene based on these 30 studies. It is noteworthy that approximately a quarter of all patients with LCA likely have responsible mutations in genes that remain unidentified.

**Table 3:** Proportions of patients with mutations in identified Leber congenital amaurosis genes.

**Study/Gene**	**AIPL1**	**CEP290**	**CRB1**	**CRX**	**GUCY2D**	**IMPDH1**	**LRAT**	**RDH12**	**RPE65**	**RPGRIP1**	**TULP1**	**Total patients screened**
Marlhens et al. 1997 [30]									1 (8%)			12
Morimura et al. 1998 [11]									7 (16%)			45
Freund et al. 1998 [31]				2 (3%)								74
Swaroop et al. 1999 [32]				1 (11%)								9
Lewis et al. 1999 [33]											0 (0%)	25
Dharmaraj et al. 2000 [34]				2 (2%)	6 (6%)				3 (3%)			100
Sohocki et al. 2000 [35]	11 (6%)											188
Lotery et al. 2000 [36]				5 (3%)	11 (6%)				12 (7%)			176
Silva et al. 2000 [37]				2 (3%)								74
Perrault et al. 2000 [38]					24 (20%)							118
Thompson et al. 2000 [39]									13 (11%)			114
Ruiz et al. 2001 [24]							0 (0%)					38
Zhang et al. 2001 [40]				1 (4%)								27
Rivolta et al. 2001 [10]				2 (3%)								62
Dryja et al. 2001 [16]										3 (5%)		57
den Hollander et al. 2001 [41]			7 (13%)									52
Simovich et al. 2001 [42]									8 (8%)			98
Gerber et al. 2001 [43]										8 (6%)		142
Sohocki et al. 2001 [44]	3 (11%)			1 (4%)								27
Sitorus et al. 2003 [45]	1 (5%)				0 (0%)				2 (10%)			21
Hanein et al. 2004 [46]	6 (3%)		18 (10%)	1 (1%)	38 (21%)				11 (6%)	8 (4%)	3 (2%)	179
Dharmaraj et al. 2004 [47]	26 (9%)											303
Perrault et al. 2004 [25]							0 (0%)	8 (4%)				179
den Hollander et al. 2004 [48]			0 (0%)									44
Zernant et al. 2005 [49]	16 (6%)		11 (5%)	3 (1%)	24 (12%)				5 (2%)	10 (5%)		205
Booij et al. 2005 [50]			1 (11%)		1 (11%)				2 (22%)			9
Bowne et al. 2006 [51]						2 (8%)						24
Yzer et al. 2006 [29]	3 (5%)		9 (15%)	0 (0%)	6 (10%)			0* (0%)	1 (2%)	0 (0%)		58*
den Hollander et al. 2006 [52]		16 (21%)										76
Sweeney et al. 2007							0 (0%)					97**
Totals	66/981	16/76	46/547	20/991	110/866	24-Feb	0/314	8/201	65/1017	29/641	3/204	
Overall Frequency	7%	21%	8%	2%	13%	8%	0%***	4%	6%	5%	1%	75%****

The table includes surveys of patients described as having Leber congenital amaurosis (LCA) or early-onset retinal dystrophy. Patients with a detected mutation in one or both alleles in a given gene are considered to have disease due to that gene. We exclude reports of surveys of fewer than nine patients; e.g., we exclude the reports of single patients with mutations in *CRX* [26] and *LRAT* [5]. We also do not include papers for which pathogenic mutations could not be distinguished from nonpathogenic rare variants (e.g., the survey of *CRB1* by Lotery et al. [27]) or papers that did not specify the number of unrelated LCA or early-onset retinal dystrophy patients screened (e.g. Thompson et al. [4] and Galvin et al. [28]). The single asterisk indicates that in the study by Yzer et al. [29], 58 patients were evaluated for the listed genes except for *RDH12*, for which only 22 were evaluated. Double asterisk indicates the 97 patients include 82 patients in our current screen and 15 LCA patients previously solved for mutations in other genes and not included in our screen. Triple asterisk indicates the percentage of LCA patients with *LRAT* mutations is indicated as zero but is definitely higher (see text). Four asterisks indicate the total proportion of patients with mutations identified.

Table 3 lists three studies that analyzed *LRAT* in a combined total of 314 LCA patients. No patients with *LRAT* mutations were found. Nevertheless, there are 4 reported LCA patients with pathogenic *LRAT* mutations [4,5]. Thompson et al. [4] reported 3 unrelated LCA patients with *LRAT* mutations, but this paper is not included in Table 3 because the number of LCA patients evaluated was not provided. Senechal et al. [5] reported one patient with LCA due to *LRAT*, but this report was also not included in Table 3 because only one LCA patient was evaluated. Based on these reports and the studies such as ours that found no LCA patients with *LRAT* mutations, it is clear that *LRAT* mutations account for a very small percentage of LCA cases. In Figure 1, we have arbitrarily set the percentage at 1%.

Our not finding mutations in a set of 190 unrelated patients with ARRP indicates that *LRAT* is not a common cause of that disease as well. Furthermore, Ruiz et al. (2001) [24] found no pathogenic mutations in 91 patients with retinitis pigmentosa, 58 patients with cone-rod dystrophy, or 93 patients with age-related macular degeneration. Senechal et al. (2006) [5] also found no pathogenic *LRAT* mutations in 134 patients with simplex or multiplex retinitis pigmentosa or in 82 patients with flecked retinal dystrophy. Based on our large survey together with the surveys in the literature, one may confidently conclude that *LRAT*-caused retinal degeneration is very rare. This rarity can have implications for the development of therapies for patients with this condition. Efficacy studies for proposed therapies may have to be based on results from only a few patients or based on results from similar therapies developed for patients with other defects in the vitamin A cycle affecting the RPE.
